# Haemorrhagic retroperitoneal paraganglioma initially manifesting as acute abdomen: a rare case report and literature review

**DOI:** 10.1186/s12893-020-00953-y

**Published:** 2020-11-30

**Authors:** Yanliang Yang, Guangzhi Wang, Haofeng Lu, Yaqing Liu, Shili Ning, Fuwen Luo

**Affiliations:** 1 Department of Hepatobiliary Surgery, The First Affiliated Hospital of Yangtze University, Hangkong Road, Jingzhou City, Hubei Province People’s Republic of China; 2 Department of General Surgery, The Second Hospital of Dalian Medical University, Zhongshan Road, Shahekou District, Dalian City, 116023 Liaoning Province People’s Republic of China

**Keywords:** Retroperitoneal paraganglioma, Pheochromocytoma, Haemorrhage, Acute abdomen, Diagnosis, Treatment

## Abstract

**Background:**

Paragangliomas (PGLs) are extremely rare neuroendocrine tumours arising from extra-adrenal chromaffin cells. PGLs are clinically rare, difficult to diagnose and usually require surgical intervention. PGLs mostly present catecholamine-related symptoms. We report a case of Acute abdomen as the initial manifestation of haemorrhagic retroperitoneal PGL. There has been only one similar case reported in literature.

**Case presentation:**

We present a unique case of a 52-year-old female with acute abdomen induced by haemorrhagic retroperitoneal PGL. The patient had a 5-h history of sudden onset of serve right lower quadrant abdominal pain radiating to the right flank and right lumbar region. Patient had classic symptoms of acute abdomen. Abdominal ultrasound revealed a large abdominal mass with a clear boundary. A Computed Tomography Angiography (CTA) of superior mesenteric artery was also performed to in the emergency department. The CTA demonstrated a large retroperitoneal mass measured 9.0 × 7.3 cm with higher density inside. A provisional diagnosis of retroperitoneal tumour with haemorrhage was made. The patient received intravenous fluids, broad-spectrum antibiotics and somatostatin. On the 3^rd^ day of admission, her abdominal pain was slightly relieved, but haemoglobin decreased from 10.9 to 9.4 g/dL in 12 h suggesting that there might be active bleeding in the abdominal cavity. Thus, we performed a midline laparotomy for the patient. Haemorrhage was successfully stopped during operation. The retroperitoneal tumour with haemorrhage was completely removed. The abdominal pain was significantly relieved after surgery. The patient initially presented with acute abdomen instead of catecholamine-related symptoms. The diagnosis of retroperitoneal PGL with haemorrhage was finally confirmed by postoperative pathological and immunohistochemical results. The postoperative course was uneventful. At the 1-year follow-up visit, no tumour recurrence was observed by Single Photon Emission Computed Tomography. A literature review was performed to further understand and analyse the aforementioned disease.

**Conclusion:**

Acute abdomen as the initial manifestation of haemorrhagic retroperitoneal paraganglioma is extremely rare. Abdominal Computed Tomography is essential to locate the lesion and differentiate between other causes of acute abdomen. PGLs are hypervascular tumours. We should be aware that ruptured retroperitoneal PGL with massive bleeding could be life threatening and require emergency laparotomy.

## Background

Paragangliomas (PGLs) are rare neuroendocrine tumours originating from extra-adrenal chromaffin cells. PGLs are located along the autonomic nervous system, especially in the retroperitoneum around the organ of Zuckerkandl. Pheochromocytomas (PCCs) arise from the adrenal medulla [1]. The incidence of PGL is estimated to be 3 per million individuals and has increased significantly over the past two decades [2, 3]. PGL usually presents catecholamine-related symptoms including hypertension, sweating, palpitations, headache and anxiety. In addition to catecholamine-related symptoms, PGL can also be asymptomatic until reaching a large size or having complications, for example, cardiac arrhythmias [4]. Biochemical testing plays an important role in diagnosis of PGL and aims to confirm the over production of catecholamines or metanephrines [5]. Imaging is widely used to indicate lesions suspected for PGL, assess regional spread or multifocality and exclude metastasis [6, 7]. The definitive treatment for PGL is surgical resection [8].

Acute abdomen is a clinical syndrome characterized by acute abdominal pain that is severe, rapid in onset and localized or generalized [9]. Acute abdomen induced by ruptured PCC with haemorrhage is more frequently reported in the literature [10]. While, haemorrhagic retroperitoneal PGL initially manifesting as acute abdomen is extremely rare. There has been only one similar case reported in literature [11].

## Case presentation

A 52-year-old Chinese female was admitted to the emergency department for 5-h history of acute onset of right lower quadrant abdominal pain accompanied with nausea and vomiting. The pain was crampy, increasing in intensity, and radiated to the right flank and right lumbar region. No aggravating or relieving factors were noted. She had no headache, palpitation, sweats and hypertension. There was no significant relevant past medical history. The patient had received an open appendectomy at the age of 25 and a left ovarian cystectomy at the age of 27. She also had right salpingectomy due to an ectopic pregnancy at the age of 38. Physical examination of the abdomen showed mild tenderness in the right lower quadrant. The following laboratory data were observed: a hemoglobin concentration of 10.9 g/dL, leukocytes 14.49 × 10^9^/L, neutrophilic granulocyte 87.10%. Abdominal ultrasound showed a large abdominal mass with a clear boarder. Blood flow could be found inside it. The patient suffered from an intolerable acute abdomen. The superior mesenteric artery embolus and abdominal aortic aneurysm were also suspected. Computed Tomography Angiography (CTA) was further done to identify nature of the retroperitoneal tumour and make differential diagnosis.

**Fig. 1:**
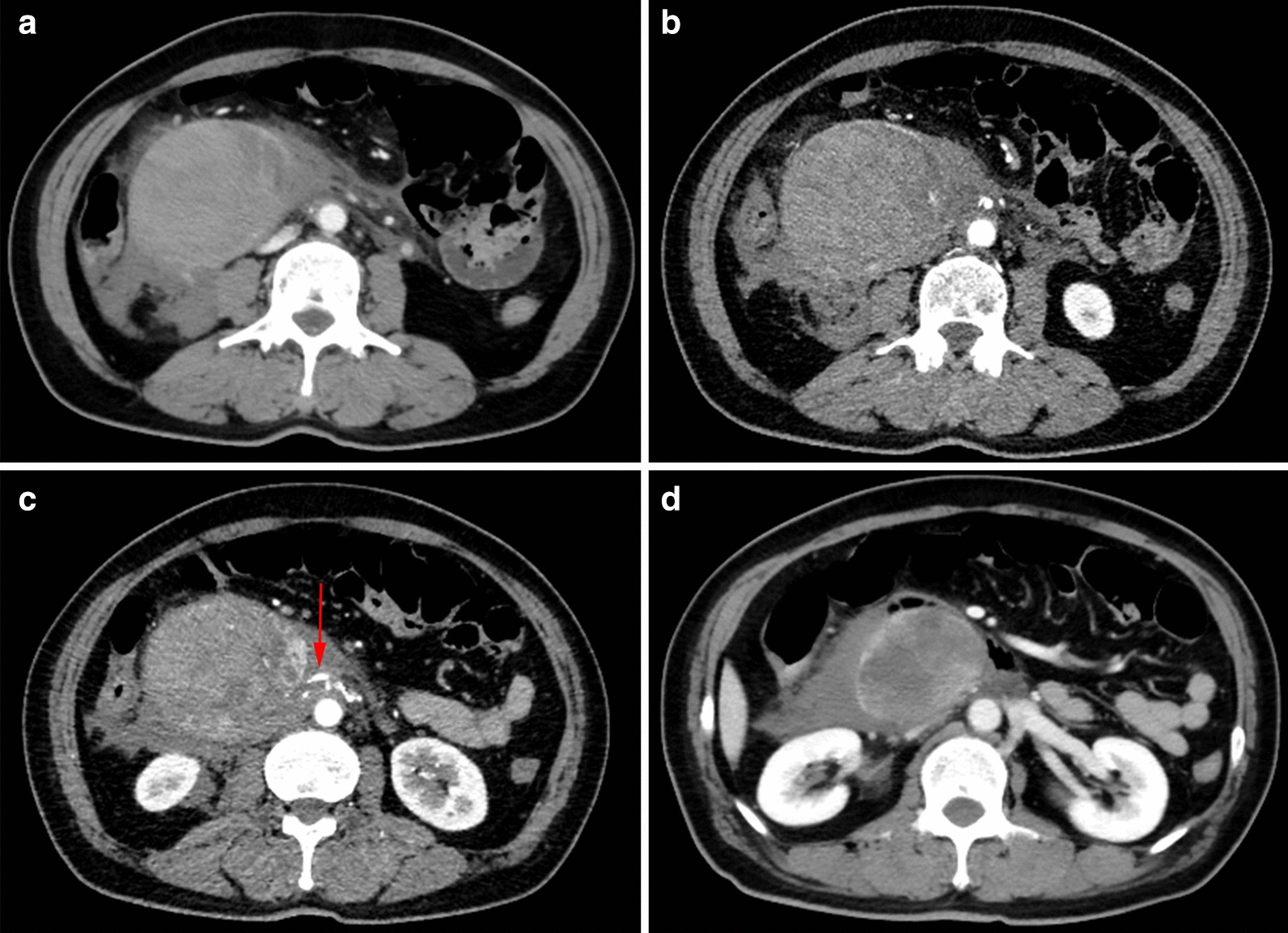
Computed Tomography Angiography (CTA) of superior mesenteric artery. The enhanced CT scan of the abdomen showed a retroperitoneal mass located posterior to the 3^rd^ part of duodenum and below the head of the pancreas. The retroperitoneal mass had a slightly increased but non-uniform density shadow at venous phase (**a**). No enhancement appearances at arterial phase (**b**). The abdominal aorta sent out small branches to supply the tumor (arrow) (**c**). There was plentiful effusion around the tumor (**d**)

According to the CTA of superior mesenteric artery, a large retroperitoneal mass measured 9.0 × 7.3 cm was located posterior to the 3rd part of duodenum and below the head of the pancreas. The retroperitoneal mass had a slightly increased but non-uniform density shadow at venous phase. The density of retroperitoneal mass showed no enhancement appearances at arterial phase (Fig. 1a, b). The abdominal aorta sent out small branches to supply the tumor (arrow) (Fig. 1c). There was plentiful effusion around the tumor (Fig. 1d).

The patient was admitted to our department. From these physical and radiographic findings, we diagnosed a retroperitoneal tumour with haemorrhage. The pain was crampy and intolerable. Emergency surgery was initially recommended after admission. But the patient wished to avoid operation and chose conservative treatment. She received treatment of intravenous fluids, hemagglutinase, and somatostatin. Antibiotic was also used to prevent abdominal infection. It was quite risky that the patient initially refused to follow our advice. We continuously assessed the pain degree of patient and measured haemoglobin concentration. Measurement of tumour markers, including AFP, CA 19-9, and CEA, were also finished. The levels of tumour markers were within the normal range. On the 3^rd^ day of admission, her abdominal pain was slightly relieved, but hemoglobin decreased from 10.9 to 9.4 g/dL in 12 h, suggesting the presence of active bleeding in the abdominal cavity. Hence, she was persuaded to have immediate surgical intervention and underwent an exploratory laparotomy.

**Fig. 2:**
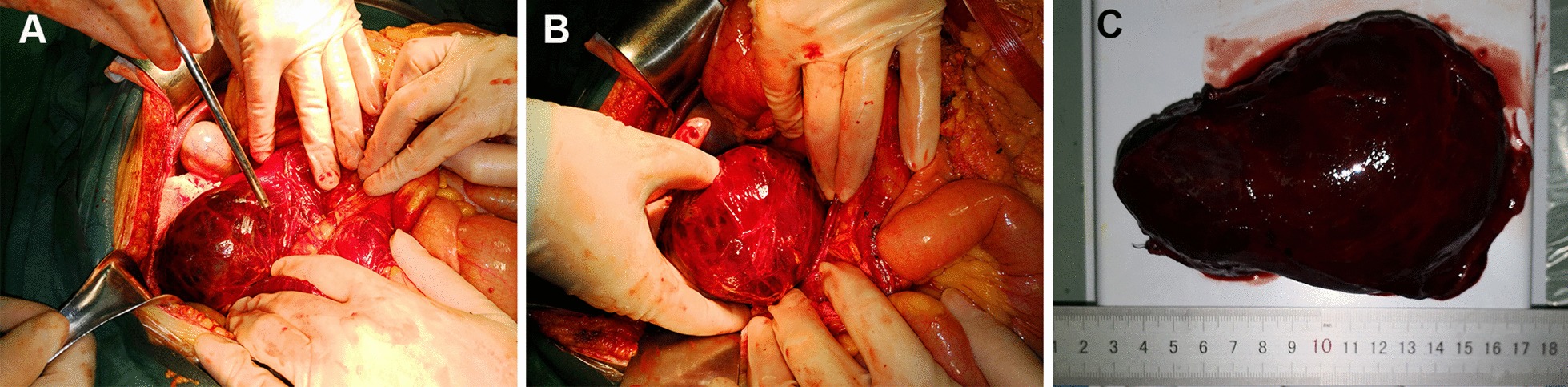
Intraoperative findings. A scarlet red retroperitoneal tumour located between the colonic hepatic flexure, the 2nd to 3rd parts of the duodenum, pancreatic uncinate process and the abdominal aorta. The tumour had a complete capsule and dense peritumoural adhesions to the surrounding blood vessels (**a**). The tumour was completely removed (**b**). The tumour body measured 12.5*8*3 cm. Haemorrhage was observed inside the tumour body, and old blood clots could be seen (**c**)

Upon the surgical exploration: A scarlet red retroperitoneal tumour was found between the colonic hepatic flexure, the 2nd to 3rd parts of the duodenum, pancreatic uncinate process and the abdominal aorta. The tumour had a complete capsule and dense peritumoural adhesions to the surrounding blood vessels (Fig. 2a). The feeding vessels of the tumour were carefully stripped and ligated to stop bleeding. Manipulation of the tumour was light to avoid rupture of the capsule. We completely removed the tumour (Fig. 2b). We also performed a regional lymph node dissection for preventing recurrence of malignant tumour. The tumour body measured 12.5*8*3 cm. Haemorrhage was observed inside the tumour body, and old blood clots could be seen. (Fig. 2c). The operative time was about 2 h. An estimated blood loss of 600 ml was noted intraoperatively. Haemorrhage was successfully stopped. After achieving haemostasis, we placed a multi-channel intraperitoneal drainage tube in a suitable position in the excised area. The patient didn't have any hypertension or tachycardia crisis during the surgery.

**Fig. 3:**
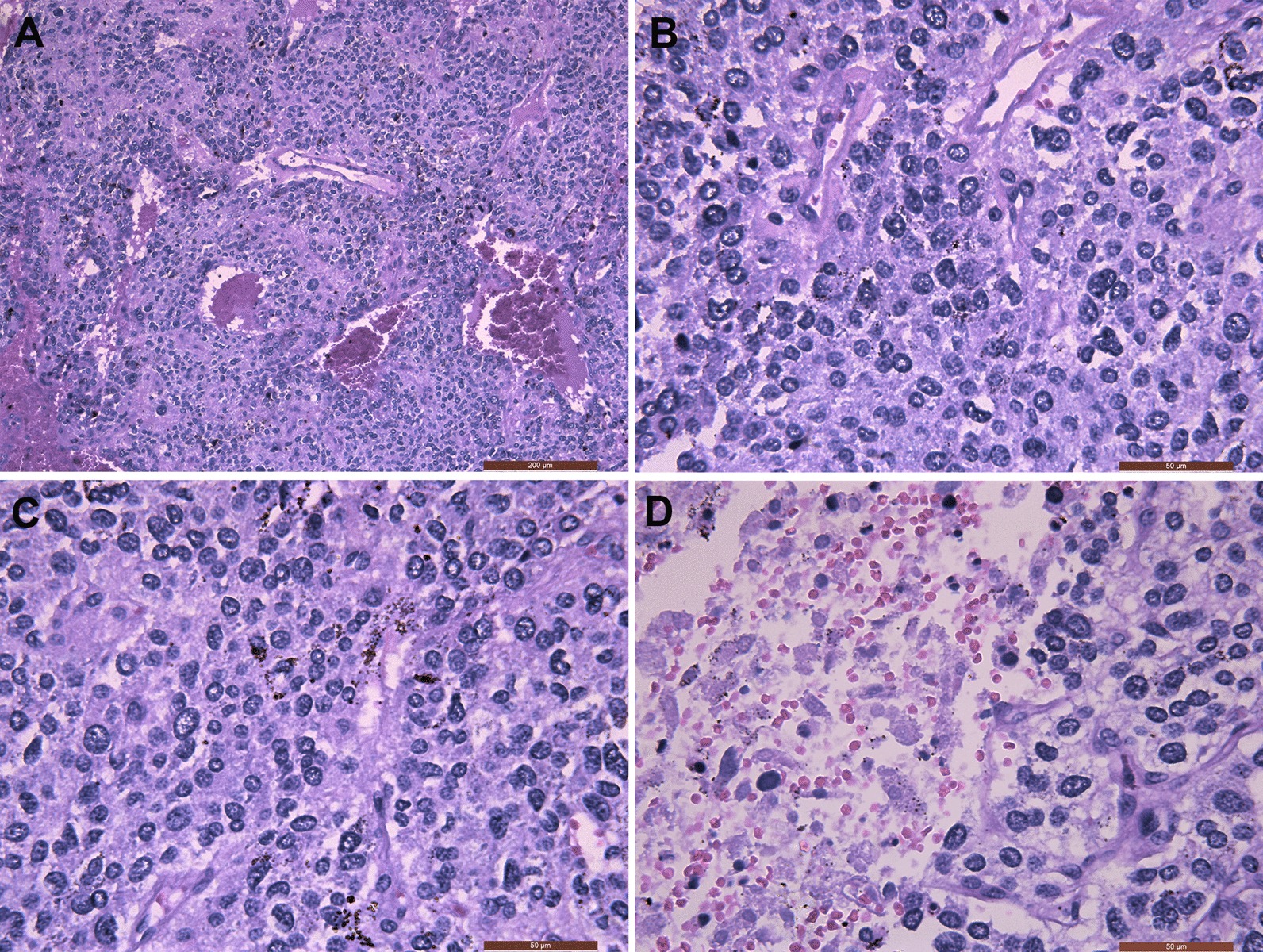
Histological features. This retroperitoneal paraganglioma showed a characteristic ‘Zellballen’ architecture in the H&E sections (haematoxylin and eosin staining, H&E staining). The tumour cells were nest-like, arranged in a cribriform pattern and separated by a rich capillary network (H&E staining, **a** HE, × 100; **b** HE, × 400). The tumour had cells of different sizes and an abundant blood supply. Nuclei were deeply stained (H&E staining, magnification × 400) (**c**). Bleeding and necrosis were seen within the tumour (H&E staining, magnification × 400) (**d**)

Histopathological assessment of the retroperitoneal tumour demonstrated a scarlet red tumour with a complete capsule containing dark blood clots. Microscopically, the tumour cells were nest-like, arranged in a cribriform pattern, surrounded by a rich network of delicate arborizing vasculature, and showed a characteristic ‘Zellballen’ architecture in haematoxylin and eosin (H&E) staining (Fig. 3a, b). The cells had different sizes, and their nuclei were deeply stained. Although the tumour adhered closely to blood vessels, no actual vascular invasion was seen (Fig. 3c). There was necrosis of the tumour body around the bleeding area (Fig. 3d). There was no metastasis in regional lymph nodes.

**Fig. 4:**
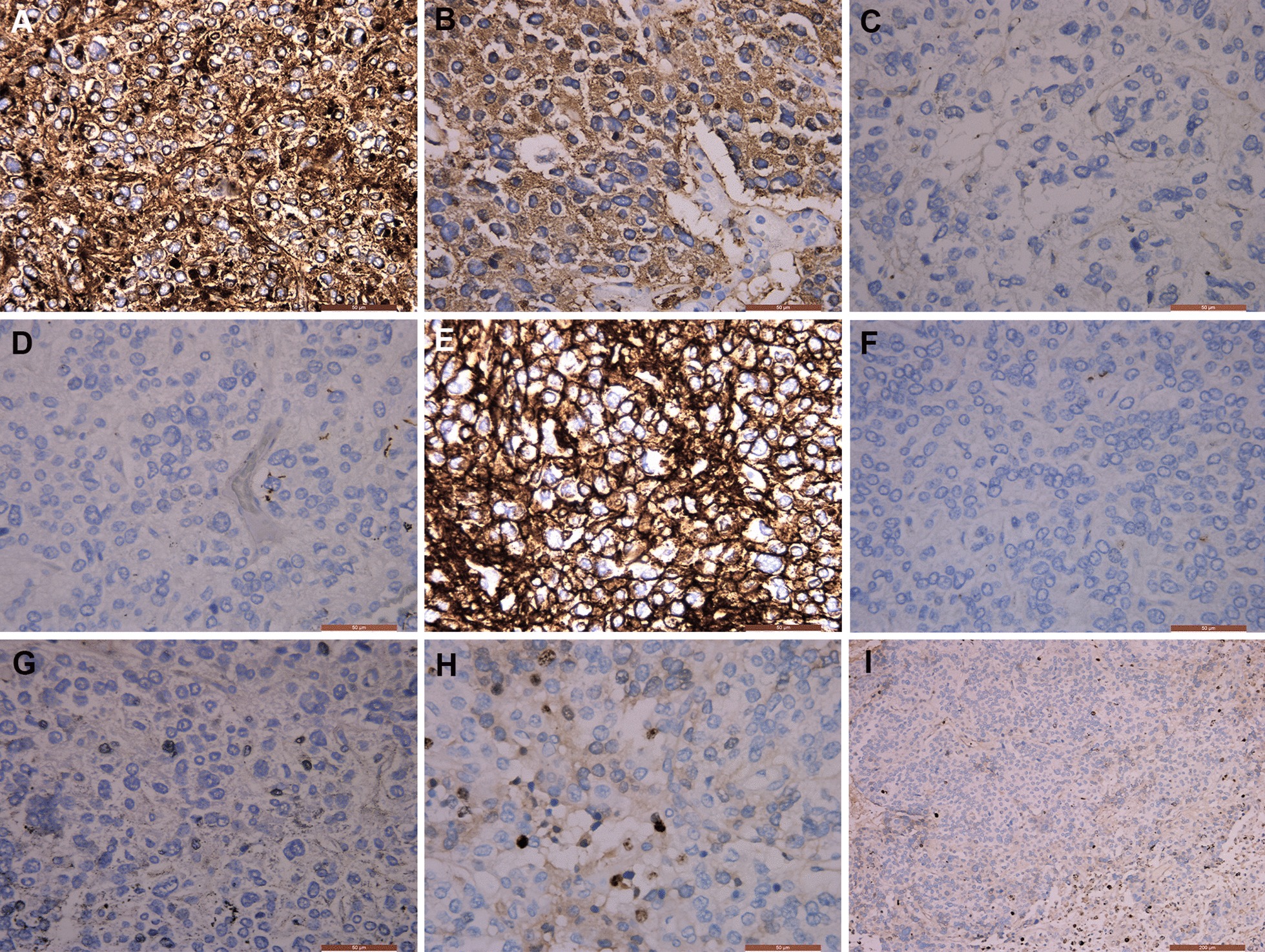
Immunohistochemistry of the retroperitoneal tumour. Immunohistochemical staining of the retroperitoneal tumour revealed the following staining results: CgA (+) (**a**), Syn (+) (**b**), DOG1 (−) (**c**), S-100 (−) (**d**), CD56 (+) (**e**), AE1/AE3 (−) (**f**), CD117 (+) (**g**), and Ki-67 (10%, +) (**h**, **i**). (**a**, **b**, **c**, **d**, **e**, **f**, **g** and **h**: × 400 magnification. **i** × 100 magnification). The tumor cells showed diffuse and strong expression of Cg A, Syn and CD56. The Ki67 index was approximately 10%

Immunohistochemistry (IHC) revealed the following profile: chromogranin A (CgA) (+), synaptophysin (Syn) (+), DOG1 (−), S-100 (−), CD56 (+), AE1/AE3 (−), CD117 (+), and Ki -67 (10%, +) (Fig. 4a–i).

Patient received antibiotic treatment for 5 days. Considering that intraoperative blood loss and high bleeding risk after surgery, she received 4 units (200 ml each) of PRBC transfusions and 640 ml fresh frozen plasma on the first postoperative day. On postoperative day 10, she was discharged, tolerating a regular diet with no further complains. At the 1-year follow-up visit, serum metanephrines of the patient were in normal range and no tumour recurrence was observed by Single Photon Emission Computed Tomography.

## Discussion and conclusions

A literature review was conducted by PubMed, Web of Science, and OVID. The documentation retrieval was identified with the key words of “paraganglioma” and “acute abdomen” through June 2020. There were few reports and acute abdomen were mostly induced by ruptured PCC. Only one case of haemorrhagic retroperitoneal PGL initially manifesting as acute abdomen was reported [11]. PGLs are rare chromaffin cell tumours arising from extra-adrenal paraganglia. It is well recognized now that all PGL/PCCs have some metastatic potential in accordance with the 2017 WHO classification of endocrine tumours [1]. A concept of risk stratification is used to describe the metastatic potential of PCCs and PGLs [12].

PGL can be further divided into sympathetic and parasympathetic types according to their clinical and biological characteristics [13]. Sympathetic PGLs account for 80% of PGLs and Parasympathetic PGLs account for 20% of PGLs. Sympathetic PGLs arise from the sympathetic nerves in the thorax and abdomen. 85% of Sympathetic PGLs locate below the diaphragm, especially in the retroperitoneum around the organ of Zuckerkandl. The organ of Zuckerkandl is a kind of chromaffin tissue located around the abdominal aorta, the inferior mesenteric artery, the beginning of the renal artery and the bifurcation of the abdominal aorta. Parasympathetic PGLs originate from the parasympathetic nervous ganglions, usually in the head and neck region [14–16].

PGLs are usually characterised by catecholamine-related symptoms and also rarely asymptomatic until reaching enormous size or having complications. PGLs mostly present with catecholamine-related symptoms and signs referable to hypersecretion of catecholamines and their metabolites [17]. Patients with PGLs usually have persistent or paroxysmal hypertension, sweating, palpitations, headache, anxiety and so on. Sudden catecholamine release may be life-threatening, causing pulmonary oedema, alveolar haemorrhage and cardiovascular accidents such as cerebral haemorrhage, hypertensive crisis, cardiac arrhythmias and myocardial ischaemia [18–21]. Parasympathetic PGLs, calling head and neck PGLs, are non-functional without catecholamine-related symptoms. Parasympathetic PGLs and part of sympathetic PGLs are initially asymptomatic and gradually have various compression-related symptoms owing to their different location and size [22]. Our patient had sympathetic PGL and was asymptomatic until the acute abdomen was induced by haemorrhagic retroperitoneal PGL.

Genetic testing is advised for patients with PGLs to find out genetic mutations causing the disease. The revolutionary genetic advances have already improved understanding and made more discoveries of PGL [23, 24]. Currently, 20 susceptibility genes for PGL and PCC have been found. The detected germline mutations, including SDHA, SDHB, SDHC, SDHD, SDHAF2, HIF2α, MAX, VHL, TMEM 127 and RET, are associated with PGLs [23, 25]. Patients, who have a positive family history or are younger than 50 years, are strongly recommended to complete genetic testing [26]. Our patient was 52-year-old and had a negative family history strongly of PGL. The pathological result also showed no lymphatic metastasis. We also strongly advised patient to perform the genetic testing. However, the patient refused to take genetic testing.

Biochemical testing should ascertain whether oversecretion of catecholamines or metanephrines exist [27]. In both plasma and urine, free metanephrines are specific markers of chromaffin tumours and superior to catecholamines, since some of these tumours do not release catecholamines but continuously produce them [28]. Catecholamines are metabolized within PGL cell to metanephrines or methoxytyramine, which are continuously released from PGLs [29]. Measurement of metanephrines in plasma has better sensitivity and specificity than metanephrines in 24-h urine, as collecting 24-h urine samples is more challenging than a single blood draw [30, 31]. Urinary vanillylmandelic acid (VMA) is the least sensitive diagnostic test [32]. A marginally increased (slightly above the upper limit of normal range) level of plasma or urinary metanephrines is not absolutely diagnostic of PGL, whereas a level of more than fourfold above the upper reference level is associated with almostly 100% probability of having these tumours [33]. We failed to diagnose the paraganglioma and confirm oversecretion of catecholamines or metanephrines preoperatively. Because the patient with retroperitoneal paraganglioma initially presented acute abdomen, instead of catecholamine-related symptoms.

Imaging examination for PGLs should be done simultaneously to make diagnosis and differential diagnosis. Imaging is usually performed after laboratory examination confirms the catecholamine excess. CT or magnetic resonance imaging (MRI) is necessary to promptly locate the PGLs. As for PGLs, the sensitivity of CT is lower than MRI [5, 34]. Functional imaging is widely applied in indicating for lesions suspected for PGL with indeterminate biochemical testing, assessing for local spread or multifocality and excluding the metastasis [35]. Metaiodobenzylguanidine scintigraphy (MIBG) is highly sensitive, especially when the CT and MRI results are negative or equivocal, and is particularly helpful in diagnosing extra-adrenal tumours and metastases [18, 36]. Positron emission tomography (PET) with ^18^F-fluorodihydroxyphenylalanine, ^18^F-fluorodeoxyglucose, ^18^F-fluorodopamine, ^11^C-hydroxyephedrine or ^68^Ga-labelled somatostatin analogues have lower radiation exposure and superior image quality but a higher cost than MIBG However, these PET approaches can be used as alternatives to ^123^I-MIBG or as additional procedures if ^123^I-MIBG scanning is negative [37, 38]. Our patient suffered from serve abdominal pain. The CTA played an important role in finding the retroperitoneal tumour with haemorrhage and making differential diagnosis with other causes of acute abdomen, such as superior mesenteric artery embolus, abdominal aortic aneurysm, acute appendicitis, cholecystitis and right renal stone.

Full preoperative preparations for patients with PGL are important to avoid perioperative complications, especially when biochemical testing confirms oversecretion of catecholamines. Patients should receive preoperative medications including α-adrenergic blockers, β-adrenergic blockers and calcium channel blockers if necessary. Admitted patients should receive preoperative fluids the night before surgery to increase blood volume, which prevents a rapid increase (with catecholamine release) or decrease (with a decrease in the catecholamine burden) in blood pressure [39]. Our patient did not present any symptoms and signs of catecholamines. Due to massive bleeding, she received preoperative fluids.

The standard treatment for PGL is surgical resection. PGLs rely on many kinds of operations due to their different locations. The laparoscopic surgery for abdominal PGLs has been proved to be as reliable as laparotomy. Some surgeons insist a retro-peritoneal laparoscopic surgery for suprarenal PGLs and a transperitoneal laparoscopic surgery for infrarenal PGLs [40]. However, the anatomic variations, instability haemodynamics during operation, close to major blood vessels, peripheral dense adhesions and hypervascularity of PGLs make laparoscopic resection extremely difficult [41]. Thus, conventional laparotomy for PGLs should be valued because conversion to laparotomy during laparoscopic surgery might occur [42–44]. Laparotomy is an appropriate choice for patients with huge tumours or difficult-to-approach PGL. Based on our experiences, excision of the tumour should be performed carefully to make sure that complex and multiple tumour-feeding vessels are well ligated.

To improve the outcomes of patients who cannot receive surgical treatment, palliative treatment options contain localized therapies such as radiotherapy, radiofrequency or cryoablation and systemic therapies like chemotherapy or molecular targeted therapies. The therapeutic principle goals for metastatic PGLs aim to control symptoms caused by catecholamine over secretion, improve life quality, prolong the survival time, and reduce other complications [45].

Morphologically, PGL typically consists of polygonal chromaffin tumour cells with sustentacular cells around. Moreover, tumour cells are separated by abundant capillary network and arranged nest like (Zellballen architecture). Cytological characteristics includes granular cytoplasm, prominent nucleoli, vesicular nuclei, pseudoinclusions inside nuclei and so on. There might be secondary changes like haemorrhage, haemosiderin deposition, sclerosis and pigmentation of lipofuscin or melanin. Pathologists could easily diagnose it according to its characteristics [46–48].

Immunohistochemistry could confirm the pathological diagnosis and make differential diagnosis with other microscopically similar tumours. CgA is the most specific feature and helps distinguish PGLs from other neuroendocrine tumours. PGLs are usually positive for synaptophysin (Syn), which is less specific than CgA because adrenal cortical carcinomas can also have diffused positive Syn staining [49–51]. CD56 is also an important neuroendocrine marker [14]. PGLs are usually negative for keratins. In our experience, S100 immunohistochemistry is not useful diagnostically since sustentacular cells are not rarely absent in other tumours [52]. A Ki-67 proliferation index above 3% significantly predict the malignant potential and prognosis of PGLs [53].

Our patient did not have any preoperative symptoms secondary to catecholamine secretion, such as headache, palpitations, sweats or tremor. Hypertensive crisis was not found during the exploratory laparotomy. Pathological examination showed that the tumour cells were nest-like and arranged in a cribriform pattern. Immunohistochemistry revealed a CgA (+) and Syn (+) profile. Thus, we present a case of haemorrhagic retroperitoneal PGL. The process of diagnosis and treatment of this patient was extremely complex and difficult.

Acute abdomen induced by haemorrhagic retroperitoneal PGL is highly rare, and only 1 case has been reported worldwide by 2019. Kwok-Kay Yau reported a male with right lower quadrant pain and the acute abdomen was originally misdiagnosed as acute appendicitis. Without preoperative CT or MRI of abdomen, the retroperitoneal tumour with haemorrhage was not found until laparoscopic appendectomy. The patient had to receive a second surgery after finishing abdominal CT and MRI [11]. Our case was more urgent and dangerous than case reported by Kwok-Kay Yau because hemoglobin of our patient decreased continuously from 10.9 to 9.4 g/dL in 12 h.

Acute abdomen induced by retroperitoneal lesions is extremely rare and complex. Retroperitoneal tumor accompanied with infection, hemorrhage or compression might lead to an acute abdomen. To the best of our knowledge, retroperitoneal tumors manifesting as an acute abdomen were reported, such as retroperitoneal myelolipoma, PGL, lymphangioma, hemangiopericytoma, leiomyoma, Ewing's sarcoma, lymphangioleiomyomatosis, cystic teratoma, adrenal lipoma, spontaneous renal angiomyolipoma, PCC, renal oncocytoma, renal adenoma, carcinoid tumor and so on [10, 11, 54–65].

Myelolipoma is a rare benign tumor composed by adipose tissue and hematopoietic cells. Similar to the distributions of PCCs and PGLs, myelolipomas are usually located in adrenal gland and rarely extra-adrenal [66–68]. There have been 9 cases of retroperitoneal myelolipomas mimicking an acute abdomen in the English literature since 2008. (Table 1) [65, 69–76]. Consequently, retroperitoneal PGL mimicking an acute abdomen should make differential diagnosis with retroperitoneal myelolipoma. Extra-adrenal myelolipomas are mostly present in the presacral retroperitoneum [77]. PGLs are mostly located in the retroperitoneum around the organ of Zuckerkandl. Myelolipomas are nonfunctional tumors and do not present any symptoms until reaching large size or having complications [78]. While, majority of PGLs present catecholamine-related symptoms. Myelolipomas show elements of fat composition on CT and MR. They are usually found incidentally and show well-encapsulated and heterogeneous soft-tissue masses on CT and MR [79]. The diagnostic biopsy plays an important role in confirming the diagnosis of myelolipoma and avoiding unnecessary surgical resection [70, 74]. Both retroperitoneal myelolipoma and PGL could cause abdominal pain. Making differential diagnosis between retroperitoneal myelolipoma and PGL is not challenging for clinicians because the significant difference in locations, symptoms, imaging features and pathology characteristics are helpful to distinguish these retroperitoneal lesions.

**Table 1:** Related reports of acute abdomen induced by paraganglioma and myelolipoma since 2008

Author	Year	Age/sex	Sympotems	Imaging	Diagnosic biosy	Diagnosed preoperatively	Location	Size, cm	Therpy	Pathology
Yau et al. [11]	2008	76/male	Sudden onset of right lower quadrant pain	Abdominal US, CT and MRI	No	No	Retroperitoneal space	5.5	Surgical resection	Paraganglioma
Zieker et al. [65]	2008	75/male	Abdominal pain and mild diarrhea	Abdominal CT and MRI	No	No	Inferior to the right kidney	7 × 5x7	Laparotomy	Myelolipoma
Dann et al. [69]	2008	82/female	Lower abdominal pain	Abdominal CT	No	Yes	Presacral space	4.5 × 3.5	Surgical resection	Myelolipoma
Hernández-Amate et al. [70]	2008	64/female	Vague abdominal pain	Abdominal CT	Yes	NA	Presacral space	8 × 6.5	Conservative management	Myelolipoma
Shereen et al. [71]	2009	85/female	Abdominal pain, nausea, vomiting	Abdominal CT	No	No	Presacral space	12 × 10 × 6.5	Surgical resection	Myelolipoma
Gill et al. [72]	2010	71/female	Cramping bilateral lower quadrant abdominal pain	Abdominal CT and MRI	Yes	Yes	Presacral space	NC	Surgical resection	Myelolipoma
Maria et al. [73]	2014	84/male	Longstanding pelvic pain	Abdominal CT and MRI	No	No	Presacral space	5 × 3 × 4	Surgical resection	Myelolipoma
Fourati et al. [74]	2015	40/female	Abdominal pain	Abdominal CT and MRI	Yes	NA	Presacral space	11.5 × 8.5 × 5	Conservative management	Myelolipoma
Lee et al. [75]	2016	69/female	Chronic nonspecific abdominal pain, nausea and vomiting	Abdominal CT and MRI	Yes	Yes	Presacral space	7.6	NC	Myelolipoma
Cho et al. [76]	2018	70/female	Ongoing pelvic pain	Abdominal US, CT and MRI	No	No	Presacral space	4.2 × 3.9 × 3	Surgical resection	Myelolipoma

*CT* computed tomography, *MRI* magnetic resonance imaging, *US* ultrasound, *NA* not available, *NC* Not clear

PGLs do not present only catecholamine-related symptoms but they may also be asymptomatic, such as parasympathetic and part of sympathetic PGLs. Acute abdomen as the initial manifestation of haemorrhagic retroperitoneal paraganglioma is extremely rare. Abdominal Computed Tomography is essential to locate the lesion and differentiate between other caused of acute abdomen. It is currently recognized that PGLs have some metastatic potential and are hereditary. Biochemical testing, especially for metanephrine levels, and imaging techniques are helpful in diagnosing and locating tumours. The definitive treatment for PGL is surgical resection. Full preoperative preparation could prevent life-threatening perioperative complications. PGLs are hypervascular tumours. We should be aware that ruptured retroperitoneal PGL with massive bleeding could be life threatening and require emergency laparotomy.

## Data Availability

Not applicable.

## Abbreviations

PGLs: Paragangliomas
PCCs: Phaeochromocytomas
CTA: Computed tomography angiography
VMA: Vanillylmandelic acid
CT: Computed tomography
MRI: Magnetic resonance imaging
H&E: Haematoxylin and eosin
IHC: Immunohistochemistry
CgA: Chromogranin A
Syn: Synaptophysin
RET: Rearranged during transfection
VHL: Von Hippel-Lindau
MIBG: Metaiodobenzylguanidine scintigraphy
PET: Positron emission tomography
